# Induction and isolation of tumor antigen-specific CD4+ T lymphocytes using sorting signal directed MHC class-II expression

**DOI:** 10.1186/2051-1426-1-S1-P6

**Published:** 2013-11-07

**Authors:** Christian Ellinger, Carina Wehner, Susanne Wilde, Dolores J Schendel, Slavoljub Milosevic

**Affiliations:** 1 Institute of Molecular Immunology, Helmholtz Zentrum Muenchen, German Research Center for Environmental Health, Munich, Germany

## 

The adoptive transfer of tumor-antigen specific CD8+ T lymphocytes has shown highly promising but somewhat limited benefit in tumor immunotherapy. The poor objective clinical efficacy observed in early studies has been overcome partly by integration of patient preconditioning and improved technologies for T cell receptor gene therapy. Nevertheless, in accordance with results from peptide vaccine studies, we hypothesize that further improvement in therapeutic efficacy can be achieved through addition of tumor-antigen specific CD4+ T lymphocytes. CD4+ T cells provide pivotal help for activated cytotoxic T cells, especially by reducing exhaustion upon chronic antigen stimulation, and by supporting tumor infiltration capacity. In addition, CD4+ T lymphocytes are critical for the initiation of long lasting CD8+ T cell memory and can also exert direct antitumor effector functions, even in the absence of MHC class II expression. To support a systematic evaluation of potentially beneficial effects of CD4+ T cells in the adoptive T cell transfer setting, we developed an efficient method for the activation and isolation of tumor-antigen specific CD4+ T lymphocytes. Using selected tumor/testis antigens fused to cell internal sorting signals, we were able to utililze dendritic cells transfected with in vitro transcribed RNA for the efficient induction of tumor-antigen specific CD4+ T cells. Via antibody-mediated staining of CD40-ligand (CD40L) on the surface of specifically reactivated peripheral blood lymphocytes (PBL), we successfully isolated multiple tumor-antigen specific CD4+ T cell clones with diverse MHC class II allotype restrictions. Moreover, a method for the direct identification of CD4+ T cell epitops (DEPI) enabled us to define novel MHC-II restricted epitopes within the targeted tumor/testis antigens. Currently, isolated tumor-antigen specific CD4+ T cell clones are assessed for direct and indirect effector mechanisms to determine the possible contributions of CD4+ T lymphocytes in the immunotherapy of cancer.

